# Supplementary material for: Collaborative intelligence in AI: Evaluating the performance of a council of AIs on the USMLE
Source: PLOS Digit Health. 2025 Oct 9;4(10):e0000787. doi: 10.1371/journal.pdig.0000787 (PMC12510544; doi:10.1371/journal.pdig.0000787)
Supplement: S2 File — (ZIP) [file pdig.0000787.s002.zip › S2 Data Raw data files containing unprocessed question inputs and LLM outputs including transcripts of deliberations/STEP3_Question-38-1_Type-MC-NJ.docx]

**Facilitator** **[Round 1]:** A 70-year-old woman comes to the office because of worsening tremor of her hands. The tremor has been present for most of her life and initially was mild and would occur only when she was tired or stressed. During the past month the shaking has become more noticeable and frequent. She is now embarrassed to eat with other people because of how obvious the tremor has become. The patient has been taking fluoxetine for the past 3 weeks to help her to cope with the death of her husband 2 months ago. Medical history is also remarkable for essential hypertension controlled with lisinopril and hyperlipidemia controlled with atorvastatin. Her only other medication is occasional ibuprofen for joint pain. She used to drink one to two alcoholic beverages monthly but now drinks one glass of wine daily because, she says, it reduces her tremor. She is 168 cm (5 ft 6 in) tall and weighs 70 kg (155 lb); BMI is 25 kg/m2. Vital signs are temperature 36.4°C (97.6°F), pulse 80/min, respirations 18/min, and blood pressure 130/85 mm Hg. Physical examination shows a moderate tremor of both hands that is not present at rest. Complete blood count, serum chemistry profile, and serum thyroid function tests are ordered and results are pending. Which of the following is the most likely cause of the patient's worsening tremor? (A) Adverse effect of fluoxetine therapy (B) Bereavement reaction (C) Early Parkinson disease (D) Increase in alcohol consumption (E) Mini-strokes

**AI_D:** RESTATING THE QUESTION: A 70-year-old woman who has had a lifelong, previously mild hand tremor, has seen a worsening of the condition in the past month. She has been under stress due to her husband's recent passing, and is also taking fluoxetine, a drug used to treat depression, for the past 3 weeks. Her health history also includes essential hypertension and hyperlipidemia, and she has increased her alcohol intake to daily use, claiming it reduces her tremor symptoms. A tremor is a rhythmic, uncontrollable movement that can happen in various parts of the body but is most often observed in the hands. As she waits for her complete blood count, serum chemistry profile, and serum thyroid function test results, we are to deduce the most probable cause for her worsening hand tremor. PLANNING: The potential causes for her worsening hand tremor could lie in adverse effects from her fluoxetine therapy, a physical manifestation of her bereavement, early signs of Parkinson's disease, the increase in her alcohol consumption, or due to mini-strokes. Each of these potential causes needs to be evaluated thoroughly to determine the most likely cause. CLINICAL REASONING: Fluoxetine, a selective serotonin reuptake inhibitor (SSRI), could potentially lead to tremor but it usually presents within the first few weeks of starting the medication, and this patient's tremor has been present most of her life. Bereavement could potentially lead to stress-induced tremors, but the longevity of the tremor again makes this less likely. Early Parkinson's disease typically presents with a rest tremor, not an action tremor as depicted in this scenario. Mini-strokes (transient ischemic attacks) usually would present with other neurological signs and symptoms and usually not a tremor. Finally, regular alcohol consumption can, in some patients, help to reduce the symptoms of essential tremor, a neurological disorder that causes involuntary shaking. CONCLUDING: Using clinical reasoning based on the patient's history and the properties of the different potential causes, it's most likely that the patient's worsening action tremor is related to her increase in alcohol consumption. Repeated alcohol use can lead to dependence, and when the person is not consuming it, withdrawal symptoms like increased tremors could manifest. Her daily consumption of alcohol, which she notes reduces her tremor, supports this conclusion. RESPONSE: (D) Increase in alcohol consumption is the most likely cause of the patient's worsening tremor. She has likely developed a dependence on alcohol, and her system might be reacting with increased tremors in the absence of higher alcohol levels.

**AI_E:** RESTATING THE QUESTION: The scenario presents a 70-year-old woman who has been dealing with a tremor in her hands that has worsened over time. This tremor has intensified recently, making it difficult for her to socialize during meals. Alongside other medications (including lisinopril, atorvastatin, and occasional ibuprofen), she has recently started taking fluoxetine as a coping mechanism after her husband's death. She has also increased her alcohol consumption to once daily, explaining it helps control her tremor. After a physical examination that found a tremor absent at rest, a battery of blood tests were requested. We're tasked with finding out the most probable rationale for her increased tremor frequency. PLANNING: We need to consider several possibilities, such as side effects from fluoxetine, which could affect neuromuscular control, or a physical manifestation of her emotional distress due to her bereavement. Early Parkinson's, known to present with symptoms such as tremor, is another consideration. The increase in alcohol intake could also have neurological consequences, and potential damage stemming from mini-strokes should be ruled out as well. Another factor to evaluate would be if the tremor is worse during movement or at rest. CLINICAL REASONING: In cases of tremor, it is imperative to determine the character - typically differentiating between a resting tremor, seen in cases of Parkinson's disease, or an action tremor, which is generally evident during active movement. Given this patient's tremor is noticeable while she is making active movements rather than when at rest, it is more likely to be an action tremor rather than a resting tremor associated with Parkinson's disease. Side effects from fluoxetine generally do not include worsening of tremor; plus, the tremor has been present most of her life, which suggests the cause is probably not the fluoxetine commenced three weeks ago. While bereavement can cause physical symptoms, the lifelong presence of the tremor decreases the likelihood of the bereavement reaction being the only cause of the worsening tremor. Mini strokes (or transient ischemic attacks) generally present with acute, episodic neurological symptoms that are not confined to action tremors of the hands. Hence, in this instance, the most probable cause of this patient's worsening tremor is linked to her increased alcohol consumption, which could be affecting her neuromuscular control. CONCLUDING: Based on the clinical history and symptom pattern provided, the patient's alcohol intake stands as a strong potential catalyst for her worsened tremor, as alcohol is known to affect the nervous system and can contribute to tremors, especially when used habitually. RESPONSE: So, the most probable cause for the worsening of the patient's tremor is (D) Increase in alcohol consumption.

**AI_B:** RESTATING THE QUESTION: We are asked to determine the most probable cause of a worsening hand tremor in a 70-year-old woman who has been experiencing this condition for most of her life, although the severity and frequency have escalated within the past month. She is currently taking fluoxetine for coping with bereavement, as well as lisinopril and atorvastatin for essential hypertension and hyperlipidemia respectively. Pedantically, tremor is a generally involuntary, somewhat rhythmic, muscle contraction and relaxation involving to-and-fro movements of one or more body parts. It is the most common of all involuntary movements and can affect the hands, arms, eyes, face, head, vocal folds, trunk, and legs. PLANNING: A number of factors could contribute to the pronounced tremor in this patient, which could include her new fluoxetine medication, a reaction to grieving the loss of her husband, the early signs of Parkinson's disease, an increase in her daily alcohol intake, or the result of mini-strokes. CLINICAL REASONING: The clinical picture presented here is consistent with Essential Tremor (ET) - a common neurological disorder characterized by a postural and/or kinetic tremor, affecting hands and forearms usually and it's noticeable during voluntary movements (like eating, writing). The description of this woman's long-lasting, intermittent tremor that has become more noticeable recently highly favor ET. In fact, worsening of tremor may not be uncommon in patient with ET due to several reasons, including age, cessation or reduction of alcohol (which could suppress tremor), and emotional stress. The acute emotional stress in this case could come from her husband's death. While fluoxetine (a SSRI) may occasionally cause tremor, it is more often associated with a fine, rapid tremor rather than the coarse tremor typical of ET. Bereavement reactions may exacerbate pre-existing conditions but would not likely cause a tremor. Parkinson's Disease usually starts with a slightly slower, resting tremor mostly on one side of the body. The increase in alcohol consumption can potentially lead to withdrawal-related tremor, but alcohol actually often helps in reducing the tremor in ET. Moreover, it usually would present with additional symptoms such as coordination problems, insomnia and others. Mini-strokes or transient ischemic attacks would cause a more acute clinical presentation including various neurological symptoms. CONCLUDING: After reviewing with a clinical thought process and considering this is not a resting tremor, her history of chronic tremor that worsens with emotional stress, the lack of symptoms for other possible conditions, as well as the pattern of symptoms, the more likely conclusion is that this is an exacerbation of her pre-existing Essential Tremor (ET), and not an adverse effect of fluoxetine therapy, a bereavement reaction or a result due to early Parkinson's disease, increased alcohol consumption or mini-strokes. RESPONSE: Thus, looking into the clinical picture and other factors, none of the options (A-E) seem to be the most likely cause of the patient's worsening tremor. However, based on the symptoms and history, I would suggest the most likely cause of the patient's worsening tremor is exacerbation of Essential Tremor.

**AI_A:** RESTATING THE QUESTION: So, we're trying to determine what might be causing the progressive worsening of an elderly woman's hand tremor. This condition has always been present in her life, increasing in frequency and noticeability over the past month. She's been on fluoxetine in recent weeks to deal with the grief from her husband's death and she's also on other medications for her hypertension and hyperlipidemia. Her tremor improves with a daily glass of wine, something she has started doing lately. The tremor is not evident when she's resting. We're asked to consider potential causes behind her worsening condition, such as side-effects of fluoxetine, reaction to bereavement, early Parkinson's disease, an increased consumption of alcohol or mini-strokes. PLANNING: The first assumption in order was about fluoxetine that could be causing tremors due to its side effects. However, her tremor was present long before starting the fluoxetine therapy. The reaction to her bereavement is another assumption, but usually, bereavement reactions include psychological symptoms like depression, anger, and guilt. Parkinson's disease indeed causes tremors, but they are typically resting tremors, unlike the patient's condition. Increased consumption of alcohol might lead to tremors, but the patient already stated that the alcohol helps reduce her tremor. A mini-stroke or Transient Ischemic Attack (TIA) could indeed cause tremors. But her tremor history and the lack of other motor or sensory neurologic deficits make this less likely. CLINICAL REASONING: Fluoxetine is an SSRI antidepressant drug, and while it may cause tremor as an adverse effect, it's less likely in this scenario as the tremors began long before the initiation of the drug. Even the slight increase in her alcohol intake wouldn't make her tremors worsen but rather alleviates them. Parkinson's disease often shows up with resting tremor, bradykinesia, rigidity, and postural instability, only the former being present in the patient's case. It's also worth noting that Parkinson's tremor usually starts unilaterally. Bereavement reaction and mini-strokes are less likely due to her presentation and history of symptoms. CONCLUDING: Given her past medical history, the patient's tremors enhancing after being tired or stressed, improvements in her tremors after drinking, and the worsening over time, it's most likely that her tremors fall under the umbrella of essential tremors. Essential Tremors (ET) is a neurological disease that causes involuntary shaking, typically in the hands. ET is not typically present at rest like Parkinson's tremor, and it usually involves both hands like in the patient's case. Worsening of symptoms over time is also characteristic of ET. Stress and fatigue are known to exacerbate the condition, while alcohol intake typically alleviates it temporarily, both of which check with the patient's history. RESPONSE: Considering all the above, the worsened tremor in this 70-year-old woman's hands, which were already present since long before but got exacerbated lately, seems to be caused by an Essential Tremor (ET). Therefore none of the choices (A to E) directly answer the question, but if we must choose from them, "(D) Increase in alcohol consumption" could be mentioned since the decrease in tremors with alcohol is one aspect of the case supportive of an Essential Tremor diagnosis.

**AI_C:** RESTATING THE QUESTION: The question is asking why a 70-year-old woman, who has had a lifelong history of benign tremor - predominantly in situations of exhaustion or stress - has had a sudden increase in the severity and frequency of these episodes. Notably, there has been an upswing in these symptoms in the past month, while she has been on fluoxetine for the past 3 weeks following her husband's death. Along with this, her lifestyle has had significant alterations such as increased alcohol intake, which she describes as useful in mitigating the symptoms. Her medical history includes hypertension and hyperlipidemia, managed with lisinopril and atorvastatin respectively. Furthermore, she experiences tremor when in action and not in rest. PLANNING: To answer this question, let's first generate alternate reasons that could explain the worsening of her tremor. These could include: (1) an increase in stress from recent bereavement could exacerbate a physiological tremor; (2) the drug therapy, fluoxetine, may have nervous system side effects; (3) the increase in alcohol consumption may have neurologic consequences; (4) new onset of a neurological disorder like Parkinson's disease may be beginning; or (5) mini-strokes. CLINICAL REASONING: To prioritize these assumptions clinically, let's consider Parkinson's disease and mini-strokes. Parkinson's typically presents with other symptoms such as bradykinesia, rigidity, and postural instability which are not mentioned in the vignette. The tremor in Parkinson's is a resting tremor which this patient does not have. Mini-strokes, such as those causing lacunar infarcts, typically present more acutely with other neurological deficits, so they would be less likely. The use of Fluoxetine, a selective serotonin reuptake inhibitor (SSRI), indeed has nervous system side effects such as tremor but these usually subside with continued treatment and the long history of tremor makes it less likely to be attributed only to medication. An increase in stress due to bereavement could indeed cause an increase in physiological tremor however the patient does not seem to be under the 'fight or flight' response all the time. An increase in the use of alcohol can actually lead to a decrease in tremors while the person is under the effect of alcohol. That being said, withdrawal from alcohol after regular use can lead to tremors, but it's unlikely in this patient who is consuming a relatively low amount regularly. However, the patient reported that alcohol consumption decreased the intensity of the tremor which points to a type of tremor that can be managed by sedative substances - an essential feature of essential tremor. CONCLUDING: An essential tremor is a nervous system (neurological) disorder that causes involuntary and rhythmic shaking and can affect almost any part of your body, but the trembling occurs most often in your hands especially when you do simple tasks, such as drinking from a glass. It's usually not a dangerous condition, but it progressively worsens over time and can be severe in some people hence the name. This is further supported by no alterations in metabolism or side effects from her current medications which can affect the central nervous system and cause tremor. RESPONSE: Thus, the cause of the patient's worsening tremor is not related to the options provided in this case - adverse effect of fluoxetine, bereavement reaction, early Parkinson disease, increase in alcohol consumption, or mini-strokes, but suggests a worsening of her long-standing essential tremor, which can be exacerbated by factors like stress, fatigue, caffeine and certain medications. It's also important to note differentiating essential tremor from other causes is important as treatment plans could differ.

**Facilitator** **[Round 2]:** SUMMARIES: AI_A: The council member believes that the woman's worsening tremor is due to Essential Tremor (ET), a neurological disease that causes involuntary shaking. They argue that the tremor was present before the fluoxetine therapy, and the alcohol intake actually alleviates the tremor. They also rule out Parkinson's disease and mini-strokes due to the lack of other symptoms. They suggest that if a choice must be made from the given options, the increase in alcohol consumption could be mentioned as it supports the diagnosis of ET. AI_B: This council member also concludes that the woman's worsening tremor is likely due to an exacerbation of Essential Tremor (ET). They argue that the tremor is not a side effect of fluoxetine, a bereavement reaction, or due to early Parkinson's disease, increased alcohol consumption, or mini-strokes. They believe that none of the given options are the most likely cause of the patient's worsening tremor. AI_C: The council member agrees with AI_A and AI_B that the woman's worsening tremor is likely due to Essential Tremor (ET). They argue that the tremor is not related to the options provided in the case, but suggests a worsening of her long-standing essential tremor, which can be exacerbated by factors like stress, fatigue, caffeine, and certain medications. AI_D: This council member differs from the others by suggesting that the woman's worsening tremor is likely due to an increase in alcohol consumption. They argue that repeated alcohol use can lead to dependence, and when the person is not consuming it, withdrawal symptoms like increased tremors could manifest. AI_E: The council member concludes that the woman's worsening tremor is likely due to an increase in alcohol consumption. They argue that alcohol can affect the nervous system and contribute to tremors, especially when used habitually. QUESTION FOR COUNCIL MEMBERS: While most council members agree that the woman's worsening tremor is likely due to Essential Tremor (ET), AI_D and AI_E suggest that it is due to an increase in alcohol consumption. Can you provide more evidence or reasoning to support your conclusion, and explain why you believe the other council members' conclusions are less likely?. Based on your preceding response, how would you answer the original question: [A 70-year-old woman comes to the office because of worsening tremor of her hands. The tremor has been present for most of her life and initially was mild and would occur only when she was tired or stressed. During the past month the shaking has become more noticeable and frequent. She is now embarrassed to eat with other people because of how obvious the tremor has become. The patient has been taking fluoxetine for the past 3 weeks to help her to cope with the death of her husband 2 months ago. Medical history is also remarkable for essential hypertension controlled with lisinopril and hyperlipidemia controlled with atorvastatin. Her only other medication is occasional ibuprofen for joint pain. She used to drink one to two alcoholic beverages monthly but now drinks one glass of wine daily because, she says, it reduces her tremor. She is 168 cm (5 ft 6 in) tall and weighs 70 kg (155 lb); BMI is 25 kg/m2. Vital signs are temperature 36.4°C (97.6°F), pulse 80/min, respirations 18/min, and blood pressure 130/85 mm Hg. Physical examination shows a moderate tremor of both hands that is not present at rest. Complete blood count, serum chemistry profile, and serum thyroid function tests are ordered and results are pending. Which of the following is the most likely cause of the patient's worsening tremor? (A) Adverse effect of fluoxetine therapy (B) Bereavement reaction (C) Early Parkinson disease (D) Increase in alcohol consumption (E) Mini-strokes]

**AI_A:** RESTATING THE QUESTION: The inquiry revolves around our elderly patient who has been plagued with a steadily worsening tremor that she has dealt with for the majority of her life. However, it has become notably worse over the past month. There are several potential causes to consider for her issue - alluding to fluoxetine therapy, bereavement due to the recent loss of her husband, the onset of Parkinson's disease, a new habit of increased alcohol consumption, or the result of mini-strokes. The challenge is to identify the most likely cause. PLANNING: An aging woman who has experienced a tremor for the better part of her life may have an essential tremor, a common neurological disorder. The timing of the tremor worsening (the past month) and the initiation of fluoxetine therapy (the past 3 weeks) might link the two, suggesting the tremor is an adverse effect. A bereavement reaction following her husband's death, or an increase in daily alcohol consumption could have exacerbated the tremor. The possibility of Parkinson's disease or mini-strokes contributing to the tremor also needs to be assessed. Upon evaluation, regular alcohol consumption is the only new introduction to seemingly alleviate her tremor. CLINICAL REASONING: Essential tremor is often dismissed when the patient is at rest and can become more pronounced during movements or in the presence of emotional stress. This tremor description aligns with the patient's presentation more than the other options. The increased frequency and the improved symptoms with alcohol intake, often seen in essential tremor, correlates with her lifestyle change. The bereavement due to her husband's passing could parallel the timing of her worsened symptoms, but the tremor predates her husband's demise. Fluoxetine could exacerbate a tremor but is not known to cause one. The lack of additional Parkinson's symptoms or transient ischemic attack symptoms further deemphasizes these options. Thus, although none of the choices ideally fit the likely cause - essential tremor, the increase in alcohol consumption does allude to the clinical picture of an essential tremor. CONCLUDING: In relation to the earlier tremor onset, her life changes over the past month (including alcohol consumption), the absence of rest tremor, and the lack of other symptoms associated with Parkinson's disease or mini-strokes, it leads me to believe that our patient's tremor worsening is most likely connected to her increase in alcohol consumption. Not because alcohol contributes to the tremor but because it provides a piece of the clinical context of essential tremor. RESPONSE: Therefore, among the options provided, the most likely cause of the patient's worsening tremor is (D) Increase in alcohol consumption. However, it's essential to clarify that alcohol in this context is a coping component linked to essential tremor, not the root cause of the tremor itself.

**AI_E:** RESTATING THE QUESTION: A 70-year-old woman who has been experiencing tremors for most of her life has recently noticed them getting worse. Her tremors become more noticeable when she's fatigued or stressed and they're not present when she rests. She takes several medications such as fluoxetine, lisinopril, atorvastatin, and occasional ibuprofen. She has increased her alcohol intake recently, from monthly to daily consumption, because she believes it reduces her tremor. She has no other significant medical conditions or symptoms. The question is asking what could be causing her tremors to worsen from the given options: an adverse effect of fluoxetine therapy, bereavement reaction, early Parkinson disease, an increase in alcohol consumption, or mini-strokes. PLANNING: The first assumption could be that her worsening tremor is related to her current medications or her increased alcohol consumption, as altered medication regimens, drug interactions, or alcohol use can impact neurological function and potentially worsen tremor. Another assumption could be that her worsening tremor could be due to bereavement reaction or an emotional response to her husband's death, as stress can exacerbate neurological symptoms like tremor. Another hypothesis is the onset of a new health issue, such as early Parkinson disease or mini-strokes, which could be causing her symptoms to worsen. After reviewing these assumptions, the fact that her tremor improves with alcohol consumption and worsens during stress, fatigue and absence of alcohol, a common characteristic of Essential Tremor, suggests that her worsening tremor may be more linked to alcohol withdrawal rather than an adverse effect of fluoxetine, bereavement reaction, or early neurological disorders; therefore, increased alcohol intake can be prioritized as the most likely cause. CLINICAL REASONING: Given the patient's long history of mild tremor which has recently worsened, and the symptoms indicative of essential tremor (ET) - manifesting during activity and improving with alcohol consumption - it can be deduced that the patient's tremor is not related to an adverse effect of fluoxetine. Fluoxetine side effects do not typically include hand tremors. Even though bereavement can lead to physical symptoms, there's little evidence to suggest it can significantly worsen an existing tremor. Early Parkinson's disease, while possible, lacks other common symptoms such as bradykinesia, rigidity, or resting tremor. Mini-strokes would likely present with other neurological symptoms, which the patient is devoid of. From this perspective, the tremor’s increment coincides with the patient's increased alcohol consumption, which aligns with alcohol's capacity to temporarily alleviate ET but worsen it in the long-term due to dependence and withdrawal. CONCLUDING: Considering the clinical history and the character of the tremor, it's unlikely that the increase in tremor severity is due to an adverse effect of fluoxetine therapy, bereavement reaction, early Parkinson's disease, or mini-strokes. The most likely cause of her worsening tremor seems to be the increase in alcohol consumption, which can produce a temporary relief of action tremor (common in ET), but with regular use can lead to dependence and enhance severity during withdrawal periods. RESPONSE: Based on the given clinical information, the most likely cause of the patient's worsening tremor is (D) Increase in alcohol consumption.

**AI_B:** RESTATING THE QUESTION: The elderly female patient has been experiencing a progressively worsening tremor in her hands that has been present for a large portion of her lifetime. Recently, this tremor has become noticeably worse and is now impacting her social funtions, like eating with others. She has also started taking fluoxetine, a type of antidepressant, to help with coping with her husband's death and reports that consuming a daily glass of wine seems to help lessen her tremor. Other notable health conditions include controlled hypertension and hyperlipidemia. She is of normal weight and has no concerning vital signs. The question posed asks what is the most probable reason behind her escalating tremor, given the predefined choices. PLANNING: The choices offered are whether it's an adverse effect of fluoxetine therapy, a reaction to her mourning, the onset of Parkinson's disease, a result of increased alcohol consumption, or mini-strokes. The key assumptions here are that (1) Her worsening tremor is a direct result of one of these options and (2) that the answer isn't something outside of these choices. However, it is possible that (1) her worsening tremor is not related to any of the predefined options and maybe due to an exacerbated Essential Tremor as suggested by some of the council members; (2) Her tremor can also be a result of a combination of factors which include the options provided but is primarily driven by her existing Essential Tremor. CLINICAL REASONING: The fluoxetine's side effects do not typically include tremors and these tremors were present long before the patient started the medication. Therefore, an adverse effect of fluoxetine therapy can be ruled out. The exacerbation of her tremor doesn't fit the temporal profile for a bereavement reaction since her husband passed away two months ago, but the tremor worsened only a month ago. Parkinson's disease, although it can cause tremors, usually has them at rest, unlike this patient's tremor. Associated symptoms such as rigidity, bradykinesia, and postural instability are also missing which makes Parkinson's disease less likely. Mini-Strokes may cause tremor, but would likely present additional neurological symptoms, which are absent here. Now, considering her alcohol consumption, although increasing intake may alleviate the tremor due to alcohol's GABA agonist effect, it's not typical of alcohol to cause a tremor that is only present with activity and not at rest. This could potentially suggest that her tremors are due to her underlying Essential Tremor condition, which is exacerbated by certain triggers, and not primarily by increased alcohol consumption. CONCLUDING: Considering these details, it seems reasonable to suggest that none of the provided options is the primary cause of her worsening tremor. Instead, her long-standing Essential Tremor disorder, which has been likely exacerbated by a combination of factors such as fatigue, stress, and bereavement, seem to be responsible for her symptoms. Alcohol may therapeutically nominally alleviate tremors related to ET due to its effect on the nervous system but not induce it. RESPONSE: Although the patient's increased alcohol consumption seems to alleviate her tremors, it is not the likely cause of her condition. Instead, her escalating tremors appear to be due to a worsening of her Essential Tremor condition. Therefore, while none of the options are an ideal match, (D) Increase in alcohol consumption would be the choice to consider only because it validates the typical response of ET to alcohol and not because it is the cause of her tremor.

**AI_D:** (1) RESTATING THE QUESTION: The query seeks to determine the most likely cause of a 70-year-old female patient's continuously worsening hand tremors, which have been going on for the majority of her life, but have significantly escalated over the past month. Some factors that may be influencing her condition include her recent use of fluoxetine (an antidepressant), the emotional impact of her husband's demise (bereavement), an increase in her daily alcohol consumption, her lengthy medical history (hypertension, hyperlipidemia), and her current drug regimen (lisinopril for hypertension, atorvastatin for hyperlipidemia, occasional ibuprofen for joint discomfort). A critical detail is that the tremors aren't present at rest, and get milder temporarily when she consumes alcohol. (2) PLANNING: The underlying assumption in the question is that one of the provided options is the cause of her deteriorating tremors. However, the possibility that the tremors may be due to a longstanding neurological disorder, typically Essential Tremor (ET), also exists given her lifelong history of tremors that are improved with alcohol intake. The next step is parsing through each of the given options and determining which, if any, could plausibly be causing her tremors to worsen. (3) CLINICAL REASONING: Beginning with fluoxetine, this drug does have some known neurological side effects, but tremor exacerbation is not frequently associated with it. The patient's bereavement reaction could plausibly cause stress-induced tremor augmentation. Still, the continuation of tremor progression despite initiating fluoxetine suggests that this isn't the root cause. Parkinson's disease could be a possibility, given the patient's age and the presence of tremors. However, the lack of tremor at rest and relief with alcohol intake diverge from the typical presentation. Hypothetically, the increased alcohol consumption could, on the one hand, be causing the tremors to worsen due to withdrawal effects when she's not drinking, but it also acts to reduce her tremors temporarily as per her observation, which suggests an underlying disorder responsive to alcohol. Finally, mini-strokes or transient ischemic attacks would typically present other symptoms as well, which this patient lacks. (4) CONCLUDING: Given the patient's symptoms, the current discussion suggests that while alcohol consumption might be interacting with the patient's existing tremor condition, leading to a temporary reduction in tremors and potential subsequent worsening due to withdrawal, it is likely that she has a pre-existing neurological condition, possibly Essential Tremor. This is backed by a lifelong history of tremors, their improvement with alcohol, and lack of other neurologic signs like rest tremor or focal neurological deficits. Therefore, it seems likely that none of the provided options directly cause her deteriorating tremors. (5) RESPONSE: Considering all details given in the prompt and analyzing them in light of standard clinical reasoning, it appears that none of the given options (A. Adverse effect of fluoxetine therapy, B. Bereavement reaction, C. Early Parkinson disease, D. Increase in alcohol consumption, E. Mini-strokes) is the most likely single cause of the patient's worsening hand tremors. However, given the limited choices, 'Increase in alcohol consumption' (D) can be selected while understanding that it doesn't fully encapsulate the likely diagnosis of Essential Tremor. Further investigation is warranted in a clinical setting to confirm my hypothesis.

**AI_C:** RESTATING THE QUESTION: The query pertains to a 70-year-old woman who has been experiencing an escalating tremor in her hands. This tremor has been present for the majority of her life, though only noted during periods of tiredness or stress. Over the last month, the tremor has become more prominent and recurrent, to such an extent that the patient now feels embarrassed eating in front of others due to its visibility. Part of her medical history includes taking fluoxetine for the past three weeks to manage the emotional distress following her husband's death two months prior. She also has essential hypertension managed with lisinopril, and high cholesterol levels controlled with atorvastatin. Other aspects of her history include occasional use of ibuprofen for joint pain, and occasional alcohol consumption—though she notes that daily wine intake helps reduce her tremor. Physically, her tremor is moderate and noted in both hands. The question is requesting an identification of the likely cause behind the progression of the patient's tremor. PLANNING: The provided choices for the potential cause behind the patient's worsening tremor are (A) the effect of the medication fluoxetine, (B) reaction to bereavement, (C) the onset of Parkinson's disease, (D) an increase in alcohol consumption, and (E) multiple mini-strokes. The relevance and plausibility of each choice needs to be considered and dissected properly. For instance, is it a common side effect of fluoxetine to induce tremors? Could the stress from bereavement enhance the presence of tremors? Is it normal for Parkinson's disease to surface at this stage of life and are there any other relevant symptoms? Does increased alcohol consumption commonly induce tremors? Can mini-strokes result in frequent localized tremors? Furthermore, the fact that she has essential hypertension and hyperlipidemia is vital to consider in relation to the potential impacts of these conditions or their treatment. CLINICAL REASONING: The fluoxetine was introduced recently whereas the tremor has been present most of her life, albeit mild before. This reduces the likelihood of the tremor being an adverse reaction to the fluoxetine. A bereavement reaction could potentially enhance tremors; however, this is more likely to manifest as a transient effect, rather than causing her life-long tremor to suddenly worsen. Parkinson's disease could potentially produce tremors; however, these tremors are typically "pill-rolling" and at rest, whereas the patient's tremor is absent when at rest. The fact that her tremor diminishes with alcohol contradicts the option suggesting that increased alcohol consumption is the cause. Mini-strokes, or transient ischemic attacks, commonly manifest with a range of neurological symptoms—localized tremor is not a typical presentation. Therefore, it's plausible to hypothesize that the tremor's escalation is potentially linked to the other medication she takes or due to her pre-existing conditions—essential hypertension or hyperlipidemia—but this is not certain without further examination. CONCLUDING: The possibility of the worsening tremor resulting from an adverse effect of the fluoxetine, a bereavement reaction, early Parkinson's, increased alcohol consumption, or mini-strokes is not strongly supported by the information provided. A more likely potential source is the impact of her other medication or her diagnosed conditions. Therefore, in answering the initial question, it would be prudent to prioritize other sources not listed in the answer choices. RESPONSE: Although options (A) through (E) have been given consideration, it is probable that none of these causes are the most likely reason for this patient's worsening tremor. Her long-standing condition seems to have worsened due to other potential factors not mentioned in the choices, but possibly related to her hyperlipidemia or hypertension, or their treatment. Therefore, extra clinical scrutiny beyond these choices may be necessary to conclusively identify the cause.

**Facilitator** **[Round 3]:** SUMMARIES: AI_A: The council member believes that the patient's worsening tremor is most likely connected to her increase in alcohol consumption. However, they clarify that alcohol is not the root cause of the tremor but provides a piece of the clinical context of essential tremor. AI_B: This council member suggests that the patient's escalating tremors appear to be due to a worsening of her Essential Tremor condition. They believe that while alcohol consumption seems to alleviate her tremors, it is not the likely cause of her condition. AI_C: The council member hypothesizes that the tremor's escalation is potentially linked to the other medication the patient takes or due to her pre-existing conditions—essential hypertension or hyperlipidemia. They believe that none of the provided options is the most likely reason for this patient's worsening tremor. AI_D: This council member suggests that while alcohol consumption might be interacting with the patient's existing tremor condition, leading to a temporary reduction in tremors and potential subsequent worsening due to withdrawal, it is likely that she has a pre-existing neurological condition, possibly Essential Tremor. AI_E: The council member believes that the most likely cause of her worsening tremor seems to be the increase in alcohol consumption, which can produce a temporary relief of action tremor (common in ET), but with regular use can lead to dependence and enhance severity during withdrawal periods. QUESTION: The council members seem to agree that the patient's worsening tremor is likely related to her Essential Tremor condition, but there is disagreement about the role of alcohol consumption. Some members believe it is the most likely cause of the worsening tremor, while others see it as a coping mechanism or a factor that interacts with the existing condition. Can the council members clarify their views on the role of alcohol consumption in this case and how it interacts with the patient's Essential Tremor condition?. Based on your preceding response, how would you answer the original question: [A 70-year-old woman comes to the office because of worsening tremor of her hands. The tremor has been present for most of her life and initially was mild and would occur only when she was tired or stressed. During the past month the shaking has become more noticeable and frequent. She is now embarrassed to eat with other people because of how obvious the tremor has become. The patient has been taking fluoxetine for the past 3 weeks to help her to cope with the death of her husband 2 months ago. Medical history is also remarkable for essential hypertension controlled with lisinopril and hyperlipidemia controlled with atorvastatin. Her only other medication is occasional ibuprofen for joint pain. She used to drink one to two alcoholic beverages monthly but now drinks one glass of wine daily because, she says, it reduces her tremor. She is 168 cm (5 ft 6 in) tall and weighs 70 kg (155 lb); BMI is 25 kg/m2. Vital signs are temperature 36.4°C (97.6°F), pulse 80/min, respirations 18/min, and blood pressure 130/85 mm Hg. Physical examination shows a moderate tremor of both hands that is not present at rest. Complete blood count, serum chemistry profile, and serum thyroid function tests are ordered and results are pending. Which of the following is the most likely cause of the patient's worsening tremor? (A) Adverse effect of fluoxetine therapy (B) Bereavement reaction (C) Early Parkinson disease (D) Increase in alcohol consumption (E) Mini-strokes]

**AI_C:** 1) RESTATING THE QUESTION: This 70-year-old woman, whose tremors have gotten worse over time, has been under significant stress due to her husband's recent death. She's been taking fluoxetine to cope with her grief and has increased her daily alcohol intake in an attempt to control her tremors. We need to determine the most probable source of her escalating hand tremor. 2) PLANNING: Assumptions here include the possibility that the patient's escalating hand tremor can be linked to her increased alcohol use or to the medication she has been on - fluoxetine. Another potential cause to investigate includes worsening of an existing neurological condition, possibly Essential Tremor. An alternate assumption, however, could be that her hand tremor might be intensifying due to the combined impact of the bereavement reaction to her husband's death, her increased alcohol consumption, and the pharmacological medications she's been on. 3) CLINICAL REASONING: Initially, the correlation between the patient's tremor escalation and her increased alcohol consumption might seem obvious since she reports using alcohol to manage her tremors and it's common knowledge that alcohol can affect the nervous system. However, considering her medical history and the chronic nature of the tremor, it seems more likely that the escalating tremor is an exacerbation of a pre-existing neurological condition, such as Essential Tremor. Drinking alcohol might temporarily lessen the severity of the tremor, potentially due to alcohol's depressant effect on the nervous system, although the tremor may intensify during periods of withdrawal. Furthermore, the added stress caused by her husband's death, the initiation of fluoxetine, and potential interactions between all these factors could also contribute to the worsening of the tremor. 4) CONCLUDING: Conclusively, the patient likely has a pre-existing neurological condition that is causing the tremor, such as Essential Tremor. While the increase in alcohol consumption may temporarily reduce the tremor, subsequent withdrawal periods could lead to tremor intensification. The added stress of her bereavement reaction and the medication she's been taking cannot be overlooked as potential contributing factors to her worsening condition. 5) RESPONSE: Based on the information provided and my clinical reasoning, I would suggest that all the factors - an underlying neurological condition (Essential Tremor), increased alcohol consumption, bereavement reaction, and pharmacological treatment - are interacting to intensify the patient's hand tremor. However, if I were to choose the most likely cause among the given options, I would select (D) increase in alcohol consumption, considering the temporal relation to the increased severity of her tremor and her admission about alcohol helping her reduce the tremor.

**AI_B:** RESTATING THE QUESTION: The collective AI council, of which I am a member, is seeking to better explain our previous perspectives on the role of alcohol in this patient's situation. To clarify, the patient is a 70-year-old woman who has had a lifelong tremor in her hands, which has been worsening recently. Despite trying to manage her stress with fluoxetine following her husband's death, her tremor has noticeably increased. She also has a history of hypertension and hyperlipidemia and uses alcohol to gain temporary relief from her tremors. The council has to weigh the potential impact of the listed options, including alcohol consumption, on the worsening of her essential tremor. PLANNING: The assumption here is that one of the listed options (A to E) must be causing the sudden and significant worsening of her tremor. An alternate assumption that could be clinically considered is that her tremor could be part of her essential tremor syndrome progressing. The acceleration of her tremor could be due to a combination of factors like ageing, stress, the introduction of fluoxetine, or her increase in alcohol. We can use the original question to evaluate if alcohol consumption could be 'the most likely cause of the patient's worsening tremor.' CLINICAL REASONING: Essential Tremor is a condition characterized by postural and kinetic tremors. The tremor often starts in the hands and can occur when the person is stressed. This is consistent with the patient's condition. A clue we can use here is that she has been living with tremors for most of her life. This is significant as this indicates she likely has Essential Tremor. While alcohol might imbue some temporary relief from tremors, it is commonly acknowledged to exacerbate the severity, particularly during withdrawal. While her consumption has increased, it is unlikely to be the root reason for her worsening condition. CONCLUDING: After thoroughly considering all the assumptions and evaluating it against our clinical understanding of Essential Tremor, it seems that her increase in alcohol consumption is not the primary cause of her escalating tremor, but rather a response to it. The underlying tremor was likely already present. RESPONSE: The clinical diagnosis should not be driven by potential coping mechanisms such as the increased alcohol consumption but should focus on providing a comprehensive view of her condition. Considering the nature of Essential Tremor and how it matches the patient's long-term symptoms, an escalation in her disease condition seems to be the most likely cause for the worsening of her tremor. Therefore, based on the provided options, all of them are plausible but none seem to be the primary cause of the patient's worsening tremor.

**AI_D:** 1) RESTATING THE QUESTION: The question invites us to identify the most probable factor among the given options that could be worsening a 70-year-old woman's tremor. Her tremor condition has been present through her life, but it has recently become more apparent and frequent. She has consumed more alcohol lately, increasing from one to two drinks monthly to one glass of wine daily - she believes the alcohol helps reduce her tremor. She's receiving medication including fluoxetine, lisinopril, atorvastatin, and occasional ibuprofen. 2) PLANNING: Assumptions that could be made include that the alcohol consumption is contributing to her worsening tremor or that it's merely a coping mechanism for her. Another assumption could be that her medication is contributing to her tremor worsening. It may be important to explore whether her pre-existing essential tremor is worsening irrespective of external factors. Given the clinical context and her response to daily alcohol, I am intrigued to hypothesize that her alcohol consumption may be interacting with her essential tremor. 3) CLINICAL REASONING: In the realm of clinical neurology, alcohol is known to temporarily reduce essential tremor symptoms - this is well-documented behavior. Although alcohol consumption may give a momentary relief to the patient, it is not treating the underlying disorder and may complicate the condition. It's more likely in this case that the woman's tremor is worsening due to the natural progression of Essential Tremor itself, with alcohol being used as an ad-hoc and temporary relief measure. Regular alcohol consumption can potentially lead to tolerance and withdrawal, the latter of which can eventually worsen tremor symptoms. Her continuous alcohol use, while noticed to reduce her tremor temporarily, may be giving a withdrawal-effect-based rebound tremor exacerbation, contributing to the worsening of the symptom. 4) CONCLUDING: Using this step-by-step clinical reasoning, it is likely that the woman's escalating tremor is due to the progression of her Essential Tremor condition in interaction with the increased alcohol consumption. The chronicity and pattern of her tremor, its exacerbation in stressful states, and its temporary response to alcohol all point more towards Essential Tremor. Furthermore, the fact that she has increased her daily alcohol intake to cope with her worsening tremor, and this practice's potential implications, should be analyzed further and managed appropriately as part of her comprehensive care. 5) RESPONSE: To the question "Which of the following is the most likely cause of the patient's worsening tremor?" If I were to choose based on the given options, I would say (D) Increase in alcohol consumption seems to be a relevant factor concerning the worsening of the tremor. However, to understand this fully, it's crucial to note that it isn't the root cause. The root cause is a neurological condition, likely Essential Tremor, and the alcohol is interacting with it, temporarily reducing tremors followed by potential worsening due to alcohol withdrawal. It would be crucial to address both her neurological condition and her increasing dependence on alcohol as part of her treatment plan.

**AI_E:** 1) RESTATING THE QUESTION: The query requires us to identify the most likely cause behind the patient's deteriorating hand tremor. The individual in question is a 70-year-old woman who's had a lifelong tremor that was only noticeable when she was tired or stressed. Recently, the tremor has become more apparent and occurs more frequently. Additionally, she's been on fluoxetine for the past 3 weeks following her husband's death. She also has essential hypertension and hyperlipidemia, controlled with lisinopril and atorvastatin respectively, and occasionally takes ibuprofen for joint pain. Remarkably, she's increased her alcohol consumption from 1-2 drinks per month to a glass of wine every day and reports that it alleviates her tremor. Currently, tests such as complete blood count, serum chemistry profile, and serum thyroid function tests are underway. 2) PLANNING: Looking at the possibilities, fluoxetine adverse effect and bereavement reaction could possibly contribute to tremors, but they are unlikely to cause a lifelong tremor. Early Parkinson's disease typically involves a resting tremor, which contrasts with this woman's tremor, which is not present at rest. Mini-strokes tend to cause a sudden onset of symptoms, not symptoms that have been present for most of the patient's life and are progressively worsening. In her case, the daily consumption of alcohol seems to temporarily reduce the tremor, but she reports that it has become worse over the past month, suggesting alcohol withdrawal might make the tremor worse. 3) CLINICAL REASONING: The woman seems to have an existing long-term "lifelong" tremor that is not present at rest, which fits the pattern of an Essential Tremor. Essential Tremor is characterized by action tremor and gets worse with age, both features align with her situation. Still, the question asks for the most likely cause of the recent difficulty, and the assumption is that it is due to a new factor which is contributing to the worsening of the tremor. Given her recent increase in alcohol consumption, alcohol withdrawal is a potential consideration because alcohol has sedative effects, and withdrawal can lead to an increase in symptoms like tremors. 4) CONCLUDING: From the above reasoning, it is apparent that the patient's increased alcohol consumption and the subsequent withdrawal effects could be contributing to the worsening of her existing Essential Tremor. 5) RESPONSE: Therefore, based on the evidence and medical reasoning, the most likely cause of the patient's worsening tremor is the 'increase in alcohol consumption' (Option D), as her consumption and withdrawal could be exacerbating her existing tremor condition.

**AI_A:** RESTATING THE QUESTION: This woman, aged 70 has experienced a tremor throughout her life, worsening in the last month. Despite the calming effect of daily alcohol intake on her tremor, her case is now more pronounced and socially embarrassing. The patient is also on fluoxetine since 3 weeks for coping with her husband's death. She has essential hypertension and hyperlipidemia, managed with lisinopril and atorvastatin respectively. Occasional ibuprofen is used for joint pain. Other findings and vital signs are within normal bounds. As she is older and has a long history of tremor, several medical possibilities need to be considered. The suggested possibilities are adverse effect of fluoxetine therapy, bereavement reaction, onset of Parkinson's disease, increased alcohol consumption, or transient ischemic attacks. PLANNING: Various assumptions are possible: (1) her tremor is exacerbated by fluoxetine use, (2) grief management is contributing, (3) early stage of Parkinson's disease is manifesting, (4) escalating alcohol consumption is contributing, (5) occurrence of mini-stroke or transient ischemic attack events. This calls for scrutinizing the validity of each assumption before deducing the most probable scenario. I would prioritize the relationship between essential tremor and alcohol consumption as a clinically relevant consideration for this clinical case. CLINICAL REASONING: The patient's symptoms are suggestive of Essential Tremor (ET), a common movement disorder in older adults. Alcohol is known to reduce the severity of tremors temporarily, but can exacerbate them when the effect fades or during withdrawal. This could explain the worsening tremors over the last month coinciding with an increase in alcohol consumption. Cases of ET are often mistaken for early Parkinson's disease, but the lack of other Parkinsonian symptoms and the presence of a tremor that is not present at rest make this unlikely. Fluoxetine, while capable of causing tremor as a side effect, would not likely cause tremor exacerbations in this context. Bereavement stress could exacerbate an existing tremor, but bed tremors aren't typically a presenting symptom of a bereavement reaction. Mini-strokes or transient ischemic attacks aren't typically associated with long-term persistent tremors. CONCLUDING: The interaction of alcohol with the phenomenon of Essential Tremor appears insightful in this case. The temporary relief of symptoms with alcohol could be encouraging this 70-year-old woman to increase her alcohol consumption, which might bash back with withdrawal periods, making the tremor worse in the long run. This fits into the pattern of her life-long mild tremor fluctuating with her state of fatigue or stress and becoming distinctly pronounced over a month. RESPONSE: Therefore, based on the clinical reasoning, despite the probable neurological pathophysiology of Essential Tremor assignment, the most likely cause of this patient's escalating tremor seems to be her (D) Increase in alcohol consumption. This answer satisfies the clinical curiosity of both explaining the alternating relief and worsening of her tremor and the escalating pattern of her tremor over the last month. It is also consistent with her clinical narrative that increased alcohol intake reduces her tremor. Alcohol isn't the source of her tremor, but it very likely plays a significant role in her present escalation. It is recommended subsequent management should incorporate this factor with balancing strategies.

**Facilitator** **[Round 4]:** Summary: AI_A believes that the patient's escalating tremor is most likely due to her increased alcohol consumption. This conclusion is based on the fact that alcohol can temporarily reduce the severity of tremors, but can exacerbate them during withdrawal. AI_A also considers other possibilities such as the adverse effect of fluoxetine therapy, bereavement reaction, onset of Parkinson's disease, and transient ischemic attacks, but finds them less likely. AI_B, on the other hand, suggests that the increase in alcohol consumption is not the primary cause of her escalating tremor, but rather a response to it. AI_B believes that the underlying tremor was likely already present and that the escalation in her disease condition seems to be the most likely cause for the worsening of her tremor. AI_C suggests that all the factors - an underlying neurological condition (Essential Tremor), increased alcohol consumption, bereavement reaction, and pharmacological treatment - are interacting to intensify the patient's hand tremor. However, AI_C would select increased alcohol consumption as the most likely cause among the given options. AI_D believes that the woman's escalating tremor is due to the progression of her Essential Tremor condition in interaction with the increased alcohol consumption. AI_D suggests that the alcohol is interacting with it, temporarily reducing tremors followed by potential worsening due to alcohol withdrawal. AI_E concludes that the patient's increased alcohol consumption and the subsequent withdrawal effects could be contributing to the worsening of her existing Essential Tremor. Question to clarify disagreements: Given the differing perspectives on the role of increased alcohol consumption in the patient's worsening tremor, how can we reconcile these views? Specifically, how can we differentiate between alcohol consumption as a primary cause of the worsening tremor versus a response to an underlying condition that is worsening independently?. Based on your preceding response, how would you answer the original question: [A 70-year-old woman comes to the office because of worsening tremor of her hands. The tremor has been present for most of her life and initially was mild and would occur only when she was tired or stressed. During the past month the shaking has become more noticeable and frequent. She is now embarrassed to eat with other people because of how obvious the tremor has become. The patient has been taking fluoxetine for the past 3 weeks to help her to cope with the death of her husband 2 months ago. Medical history is also remarkable for essential hypertension controlled with lisinopril and hyperlipidemia controlled with atorvastatin. Her only other medication is occasional ibuprofen for joint pain. She used to drink one to two alcoholic beverages monthly but now drinks one glass of wine daily because, she says, it reduces her tremor. She is 168 cm (5 ft 6 in) tall and weighs 70 kg (155 lb); BMI is 25 kg/m2. Vital signs are temperature 36.4°C (97.6°F), pulse 80/min, respirations 18/min, and blood pressure 130/85 mm Hg. Physical examination shows a moderate tremor of both hands that is not present at rest. Complete blood count, serum chemistry profile, and serum thyroid function tests are ordered and results are pending. Which of the following is the most likely cause of the patient's worsening tremor? (A) Adverse effect of fluoxetine therapy (B) Bereavement reaction (C) Early Parkinson disease (D) Increase in alcohol consumption (E) Mini-strokes]

**AI_D:** To navigate through these varying perspectives, it's essential to closely consider the clinical picture of the patient. We have learned that she has long experienced tremors, which have become worse recently, even though the tremors disappear when she is at rest. This suggests Essential Tremor (ET) given that its severity progressively increases over time and it doesn't exhibit during rest. Moreover, the patient has been consuming additional alcohol, which she reports helps reduce her tremors. This aligns with the fact that alcohol can provide temporary relief to ET symptoms but may equally exacerbate tremors during withdrawal. An underlying condition may deteriorate independently, but in this case, it seems more likely that the increased alcohol consumption contributes to the worsening of her tremors, rather than merely being a response. The alcohol's double-edged nature - providing immediate relief but worsening symptoms over time - seems to align best with the patient's symptoms, over fluoxetine therapy effects or bereavement reactions. Therefore, based on a clinical reasoning process that evaluates each potential cause, it seems to make sense that the worsening of the patient's tremors is primarily caused by an increase in alcohol consumption (D), alongside the progression of her Essential Tremor. However, this doesn't exclude the possible influence of other factors such as her recent bereavement and medication. Further tests and possibly mental health counseling might be beneficial for her overall health management.

**AI_E:** RESTATING THE QUESTION: The query relates to diagnosing the most probable cause of a 70-year-old woman's intensifying hand tremor. She has had this condition all her life, but it has significantly increased over the past month, driven possibly by assorted factors such as the intake of fluoxetine, bereavement following her husband's death, Parkinson's onset, alcohol use, or mini-strokes. PLANNING: We have multiple perspectives to consider here: that the tremor’s worsening is an adverse effect of her fluoxetine medication, that it's a bereavement reaction, it might be a symptom of early Parkinson's disease, increased alcohol consumption could be the cause, or that it is due to mini-strokes. But the most significant assumption appears to be that the tremor is correlated with increased alcohol use, considering the patient's statement that her tremor subsides with alcohol intake. CLINICAL REASONING: When we perceive a woman who has been dealing with hand tremors all her life and is now seeing a significant exacerbation, we need to consider any possible changes in her life that could stimulate this alteration. Notably, this patient has amplified her daily alcohol consumption, which she believes is tempering her tremor. However, alcohol can produce a temporary abatement of tremors due to its central nervous system depressant effect. On cessation or reduction, the tremor could worsen due to withdrawal effects. So, it's plausible that her increased alcohol consumption is contributing to her tremor worsening. CONCLUDING: Given the lack of other significant findings on her history or physical exam, plus the notable alteration in her alcohol consumption pattern in parallel with the tremor escalation, it is highly plausible that the most immediate factor that has contributed to her tremor worsening is her increased alcohol consumption. RESPONSE: Considering the available given options and the patient's reported tremor history and recent changes, the most likely cause of the patient's worsening hand tremor is her increased alcohol consumption (D).

**AI_A:** RESTATING THE QUESTION: The question relates to a 70-year-old woman who has experienced tremors for most of her life. Recently, these tremors have become worse, especially in the past month, to the point where she's embarrassed to eat in public. She's started drinking alcohol daily as it helps reduce her tremor. She is presently on fluoxetine due to a bereavement and has a history of hypertension and hyperlipidemia. The query is about the possible cause of the exacerbation of her tremor. PLANNING: The presence of a tremor throughout her life suggests an underlying condition like Essential Tremor. The options given suggest we consider other alternatives like fluoxetine as a side effect, bereavement, early Parkinson's disease, an increase in alcohol consumption, or mini-strokes. Other medications might also interact with alcohol to cause the increased tremor. We can question these assumptions by examining the likelihood and clinical manifestations of each. CLINICAL REASONING: Fluoxetine can cause tremors as a side effect, but usually occurs soon after starting the medication, making it less likely in our case. Bereavement reactions could lead to increased stress and potentially worsen a pre-existing tremor, but it's a less direct cause. Parkinson's disease might present with a resting tremor, which the patient does not have. Mini-strokes would usually present with other neurological findings beyond just a worsening of a tremor. A noteworthy point is that the patient notices an improvement in her tremors after drinking alcohol. Alcohol can relieve Essential Tremor temporarily but can also worsen it during the withdrawal period, contributing to a cycle of increased consumption and worsening tremors. CONCLUDING: So, among the options provided, an increase in alcohol consumption following the death of her husband and perhaps the increased stress associated with that event appears to be the most likely primary cause of the worsening of her tremors. RESPONSE: The most likely cause of the patient's worsening tremor is an increase in alcohol consumption (D), which could be part of a cyclic pattern of relief during alcohol consumption and worsening during withdrawal periods. Other factors could contribute, but with the information provided and options given, this seems to be the most feasible cause.

**AI_C:** RESTATING THE QUESTION: In simplified terms, this query is about a 70-year-old woman who is experiencing an escalating shaking of the hands. Even though she's had this shaking for most of her life, it has become more intense over the past month. The patient also has established diagnoses of hypertension and hyperlipidemia, for which she's taking lisinopril and atorvastatin respectively. For the past three weeks, she's been on fluoxetine to help cope with the death of her husband. She's also been consuming a glass of wine daily to lessen her tremor as opposed to her previous monthly consumption. The physician is trying to ascertain the most likely cause for the worsening of her tremor from the list provided. PLANNING: To interpret this case, one must consider key facts: the patient's longstanding history of tremor, the progressive increase in intensity of the tremor, and the patient's recent increase in alcohol consumption. Furthermore, it's crucial to review the potential role of her medications and other confounding factors such as stress or grief associated with the bereavement. Consequently, alternate assumptions would include potential side effects from medications, withdrawal symptoms from increased alcohol use, the psychological impact of bereavement, the onset of Parkinson's disease or transient ischemic attacks. CLINICAL REASONING: First, considering the lifelong history of the tremor that worsens when the patient is tired or stressed, it's feasible the patient may have a condition known as "essential tremor." Essential tremor is a neurological disorder that primarily causes a shaking of the hands which usually worsens with stress and is typically bilateral. However, the recent exacerbation calls for an understanding of what induced it. Looking at the medication history, fluoxetine could potentially cause tremor as a side effect, yet in this case, it doesn't seem as probable because the tremor is an existing condition and only worsened recently. Conversely, the patient's increased consumption of alcohol appears to coincide with the worsening of her tremor symptoms. It's known that alcohol can temporarily soothe the symptoms of essential tremor but afterwards, as the body processes the alcohol and withdrawal begins, the tremor can be exacerbated. Other possible reasons, such as the outbreak of Parkinson's disease or transient ischemic attacks are less likely due to the specific characteristics of the patient's tremor (bilateral, intensifying with activity, and present for most of her life). CONCLUDING: Based on the evidence available, it appears the patient's escalating tremor is an interplay between her lifelong existing tremor probably due to Essential Tremor - which gets worse with stress, fatigue, and now possibly due to alcohol withdrawal symptoms caused by her increased consumption of alcohol. RESPONSE: The most likely cause of the patient's worsening tremor, given the provided options, would be an (D) Increase in alcohol consumption. While her underlying condition of a probable Essential Tremor remains unchanged, it's the exacerbation of symptoms likely due to the withdrawal effects from increased alcohol consumption that is notable. Even though other factors such as bereavement stress and medication side-effects might be contributing, they appear less likely in this particular case.

**AI_B:** 1) RESTATING THE QUESTION: The question is asking for the most likely cause of a 70-year-old woman's progressively worsening hand tremor that has been present for most of her life but has notably increased recently. It includes details about her life changes and medication usage, including a marked increase in alcohol consumption. The options provided encompass potential side effects of her medications, emotional responses to bereavement, neurological disorders like Parkinson's disease, the impact of increased alcohol consumption, and possible cerebrovascular events. 2) PLANNING: Considering her age, the longstanding but progressively worsening nature of her tremor, the details of her medical history and medication consumption, as well as the effects of bereavement and increased alcohol consumption, we need to analyze each potential cause. Let's generate alternative assumptions: Could fluoxetine, a selective serotonin reuptake inhibitor used for treating depression, cause a tremor? It's possible, but it would more likely lead to a generalized tremor rather than specifically in her hands. Could her worsening tremor be related to her bereavement reaction after her husband's death? It's plausible that stress could exacerbate her symptoms, but it doesn't completely explain her longer-term problem. Is it possible that she has early-onset Parkinson disease? Given the absence of resting tremor, it seems less likely. Could her increase in alcohol consumption be responsible? This seems probable- as it's known alcohol can temporarily reduce tremors but exacerbate them during withdrawal periods. Lastly, are 'mini-strokes' (transient ischemic attacks) to blame? Given the absence of other typical symptoms like speech and sensory changes, this option seems less likely. 3) CLINICAL REASONING: The woman's lifetime tremor seems to suggest a hereditary or long-standing neurological disorder such as Essential Tremor (ET), a disorder characterized by an action tremor in the hands that may become worse with stress, fatigue, and certain medications. Even though the increased consumption of alcohol can temporarily alleviate her symptoms, subsequent withdrawal can cause a rebound effect and possibly exacerbate her tremor, making her tremor more apparent and frequent in the past month. The recent bereavement may have added emotional stress, contributing to her condition's progression. The fluoxetine could potentially exacerbate her condition, but considering that her tremor has been present for most of her life, it seems unlikely that it would suddenly cause a marked worsening. 4) CONCLUDING: Considering her longstanding tremor history, which could be indicative of a disorder like Essential Tremor, the heightened emotional stress from her recent bereavement, and the potential exacerbating effect from the increased alcohol consumption, it is plausible to conclude that a complex interaction of these factors is causatively contributing to her worsening tremor. 5) RESPONSE: Therefore, while increased alcohol consumption (Option D) may be partially responsible for the patient's worsening tremor, it seems more likely a response to an already present condition that ingests response to the stress due to bereavement, tempers the tremor's symptoms temporarily but exacerbates in withdrawal. So, the most probable factor contributing to a worsened hand tremor in this case would be an escalating underlying condition, most likely Essential Tremor, in interaction with increased alcohol consumption.

**Facilitator** The consensus statement synthesizes the responses of AI_A, AI_B, AI_C, AI_D, and AI_E as follows: The question pertains to a 70-year-old woman who has experienced a lifelong tremor that has recently worsened, particularly in the past month. The woman has also increased her alcohol consumption, which she believes helps reduce her tremor. She is currently on fluoxetine due to a bereavement and has a history of hypertension and hyperlipidemia. The query is about the most probable cause of the exacerbation of her tremor. The woman's lifelong tremor suggests an underlying condition like Essential Tremor (ET), which is characterized by an action tremor in the hands that may become worse with stress, fatigue, and certain medications. However, the recent exacerbation calls for an understanding of what induced it. The fluoxetine could potentially cause tremor as a side effect, but considering that her tremor has been present for most of her life, it seems unlikely that it would suddenly cause a marked worsening. Bereavement reactions could lead to increased stress and potentially worsen a pre-existing tremor, but it's a less direct cause. Parkinson's disease might present with a resting tremor, which the patient does not have. Mini-strokes would usually present with other neurological findings beyond just a worsening of a tremor. A noteworthy point is that the patient notices an improvement in her tremors after drinking alcohol. Alcohol can relieve Essential Tremor temporarily but can also worsen it during the withdrawal period, contributing to a cycle of increased consumption and worsening tremors. The patient's increased consumption of alcohol appears to coincide with the worsening of her tremor symptoms. It's known that alcohol can temporarily soothe the symptoms of essential tremor but afterwards, as the body processes the alcohol and withdrawal begins, the tremor can be exacerbated. Therefore, among the options provided, an increase in alcohol consumption following the death of her husband and perhaps the increased stress associated with that event appears to be the most likely primary cause of the worsening of her tremors. While her underlying condition of a probable Essential Tremor remains unchanged, it's the exacerbation of symptoms likely due to the withdrawal effects from increased alcohol consumption that is notable. Even though other factors such as bereavement stress and medication side-effects might be contributing, they appear less likely in this particular case. Further tests and possibly mental health counseling might be beneficial for her overall health management.
